# Diagnostic insights into solid pseudopapillary neoplasms of the pancreas: a decade of experience with pediatric representation

**DOI:** 10.1186/s13000-025-01648-9

**Published:** 2025-04-30

**Authors:** Noura A. A. Ebrahim, Moamen O. Othman, Rasha A. Salama, Dalia Abdelfatah, Neveen S. Tahoun

**Affiliations:** 1 Oncologic Pathology Department, National Cancer Institute (NCI) - Cairo University, Cairo, Egypt; 2 Cancer Epidemiology and Biostatistics Department, National Cancer Institute (NCI) - Cairo University, Cairo, Egypt; 3 Kasr Al-Aini Faculty of Medicine, Cairo University, Cairo, Egypt; 4 Ras Al Khaimah Medical and Health Sciences University, Ras Al Khaimah, UAE

**Keywords:** Solid pseudopapillary neoplasm, Pancreas, CD99, LEF1, β-catenin, Cyclin D1, Immunohistochemistry, Pediatric pancreatic tumors, Wnt signaling

## Abstract

**Background:**

Solid pseudopapillary neoplasms (SPNs) of the pancreas are rare, low-grade malignancies that predominantly affect young females. Their diagnosis is often facilitated by a characteristic histomorphological pattern and immunohistochemical profile. However, diagnostic challenges persist, especially in pediatric and atypical presentations. Recent attention has focused on the diagnostic value of CD99 and LEF1 in distinguishing SPNs from other pancreatic neoplasms.

**Objective:**

To evaluate the diagnostic accuracy and utility of CD99 and LEF1 as immunohistochemical markers for SPNs.

**Methods:**

A retrospective analysis of 60 SPN cases diagnosed between 2015 and 2024 was performed. Histopathological features were systematically reviewed, and immunohistochemical staining for CD99, LEF1, β-catenin, Cyclin D1, PR, Ki-67 was evaluated. Immunohistochemical marker interpretation was standardized using internally validated thresholds informed by existing literature: CD99 was considered positive with ≥ 10% cytoplasmic staining exhibiting paranuclear accentuation; β-catenin positivity was defined by ≥ 5% nuclear localization; Cyclin D1 by ≥ 10% moderate-to-strong nuclear staining; and progesterone receptor (PR) expression by ≥ 1% nuclear positivity, consistent with hormone receptor evaluation guidelines.

Marker expression was statistically analyzed for their associations.

**Results:**

SPNs exhibited a strong female predilection (F:M ratio ≈ 7:1), with a mean age of 32.5 years. Pediatric cases (*n* = 4) displayed higher mean expression of CD99 (73.8%) and LEF1 (86.5%) compared to adults. CD99 showed cytoplasmic positivity with paranuclear accentuation in 96.7% of cases, while LEF1 demonstrated nuclear staining in 91.7%. β-catenin nuclear localization was observed in 95% of tumors, reflecting Wnt/β-catenin pathway activation. Cyclin D1 and PR were expressed in 90% and 88.3% of cases, respectively. Co-expression of β-catenin, CD99, LEF1, Cyclin D1, and PR was observed in 73.3% of tumors. CD99 and LEF1 inversely correlated with tumor size and proliferative activity (Ki-67), whereas Cyclin D1 and Ki-67 positively correlated with tumor size and lymphovascular invasion (LVI). Pediatric tumors generally exhibited favorable profiles, with limited evidence of LVI.

**Conclusion:**

SPNs present with distinctive immunohistochemical signatures that are critical for accurate diagnosis, particularly in morphologically ambiguous or pediatric cases. CD99 and LEF1 are highly sensitive markers that, in combination with β-catenin and Cyclin D1, enhance diagnostic precision. These findings emphasize the central role of Wnt/β-catenin signaling in SPN pathogenesis and underscore the importance of integrating clinicopathological and molecular data for comprehensive tumor assessment.

## Introduction

Solid pseudopapillary neoplasm (SPN) of the pancreas is a rare and unique epithelial tumor that was initially described by Frantz in 1959 [1]. Representing less than 2% of all pancreatic neoplasms, SPNs display a distinctive combination of solid and cystic components, with a pseudopapillary architecture. These tumors are characterized by a particular clinical and histopathological profile, which often leads to diagnostic challenges due to their overlap with other pancreatic tumors, including neuroendocrine tumors (NETs), acinar cell carcinomas, and pancreatic adenocarcinomas [2]. While SPNs most frequently affect young women in their second and third decades of life, emerging studies suggest that they can also occur in male and pediatric populations, albeit at lower frequencies. The rarity of these neoplasms, along with their unique histologic features, continues to make them a subject of extensive research and clinical interest within pancreatic pathology [3].

The morphologic features of SPNs are key to their diagnosis. These tumors typically exhibit solid and pseudopapillary architecture, along with uniform nuclei and varying degrees of cystic degeneration and hemorrhage. While these characteristics are often diagnostic in resected specimens, limited biopsies or cytological samples may not always display the classical architecture, which can complicate diagnosis. In such cases, the use of immunohistochemistry (IHC) is essential for accurate classification, as it aids in distinguishing SPNs from other pancreatic neoplasms that may share similar histologic features [2, 3].

Immunohistochemistry (IHC) plays a crucial role in the diagnostic process of solid pseudopapillary neoplasms (SPNs), particularly in distinguishing these rare tumors from other pancreatic neoplasms that may share similar morphological features. Among the immunomarkers investigated for this purpose, CD99 and LEF1 have gained significant attention for their potential diagnostic value, though they carry distinct implications in the interpretation of SPN cases. CD99, a transmembrane glycoprotein encoded by the MIC2 gene, is variably expressed in SPNs [4]. It is particularly useful in differentiating SPNs from other pancreatic tumors, such as neuroendocrine tumors and pancreatic adenocarcinomas, especially when combined with additional markers. CD99 expression in SPNs often exhibits paranuclear accentuation and can be particularly helpful in distinguishing these tumors from acinar cell carcinomas, especially when beta-catenin expression is absent. However, despite its high sensitivity, CD99 is not entirely specific, as it is also expressed in a wide range of malignancies, including small round blue cell tumors like Ewing sarcoma, desmoplastic small round cell tumors (DSRCT), CIC-rearranged sarcomas, and BCOR-altered neoplasms. These tumors can morphologically and clinically resemble SPNs, particularly in pediatric or extra-pancreatic cases, which makes relying on isolated CD99 positivity potentially misleading. Therefore, CD99 expression should always be interpreted in conjunction with other immunohistochemical markers and within the broader context of histological examination to avoid misdiagnosis [4–7].

β-Catenin is a multifaceted protein that performs critical roles within the cell, acting both as a structural component and as a regulator of gene expression. As part of adherens junctions, it links the actin cytoskeleton to cadherins, facilitating cell–cell adhesion and maintaining the integrity of tissue architecture. Beyond this structural function, β-catenin also translocates into the nucleus, where it interacts with transcription factors, notably those of the LEF- 1 family, to regulate the expression of specific genes. This dual role underscores the importance of β-catenin in responding to extracellular signals, where it transduces changes in cell adhesion into alterations in gene expression, thereby influencing cellular behavior. The regulation of β-catenin levels is essential for maintaining normal cell function, and it is tightly controlled by a network of proteins, including adenomatous polyposis coli (APC), axin, and glycogen synthase kinase 3β (GSK- 3β), which coordinate its degradation through the ubiquitin–proteasome pathway. Disruptions to this regulation, such as mutations in APC or β-catenin, result in the accumulation of β-catenin within the cell. This accumulation leads to aberrant gene transcription, primarily through its interaction with LEF- 1, contributing to tumorigenesis. The β-catenin/LEF- 1 complex plays a crucial role in driving uncontrolled cellular proliferation and tumor progression [7–10].

LEF1 (Lymphoid Enhancer-binding Factor 1) is a nuclear transcription factor that interacts with β-catenin, forming a complex that regulates gene expression within the WNT signaling pathway. This complex is integral to controlling genes involved in cell cycle regulation, such as c-Myc and Cyclin D1. In tumors driven by WNT signaling, mutations in the CTNNB1 gene, which encodes β-catenin, lead to its stabilization and nuclear accumulation. This abnormal accumulation of β-catenin is a hallmark of WNT-activated tumors, although its sensitivity as a diagnostic marker can vary. The localization of β-catenin can shift between the nucleus, cytoplasm, and membrane, making interpretation challenging. Factors such as antibody clone choice and cut-off values further complicate the reliability of β-catenin staining, posing difficulties in accurate diagnosis. Cyclin D1, a member of the conserved cyclin family, is crucial for the transition from the G1 to S phase of the cell cycle. As an oncogene, Cyclin D1 plays a significant role in malignant transformation by promoting abnormal cell growth, angiogenesis, and resistance to apoptosis. The expression of Cyclin D1, regulated by the β-catenin/LEF- 1 complex, plays a crucial role in tumorigenesis. Co-expression of LEF1, Cyclin D1, and nuclear β-catenin constitutes a robust molecular signature of Wnt-driven tumorigenesis in solid pseudopapillary neoplasms (SPNs) [11–14]. In SPNs, LEF1 expression not only provides diagnostic value but also reflects the activity of β-catenin, underscoring the dysregulation of the β-catenin/LEF- 1 signaling pathway. This highlights the critical role of this pathway in the molecular mechanisms driving SPNs and potentially other malignancies. The interaction between β-catenin and LEF- 1 is essential for tumorigenesis, offering valuable insights into the complex molecular networks that regulate cancer progression [11–14].

Beta-catenin, Cyclin D1, CD99, and LEF1 have been implicated in the pathogenesis of solid pseudopapillary neoplasms (SPNs), but their correlation with each other and their role in diagnosis remains poorly understood. Cyclin D1 regulates the cell cycle, with overexpression observed in SPNs, while the relationship between Cyclin D1, CD99, and LEF1 has not been fully explored. Further research is needed to clarify these associations.

This study aims to investigate the diagnostic utility of CD99 and LEF1 in SPNs, particularly in underrepresented groups such as pediatric patients. CD99 may serve as a supportive marker, though its expression in histologic mimics requires caution. LEF1, in contrast, may be a more specific and relevant marker for SPNs. Despite their potential, data comparing their diagnostic performance across diverse demographics is limited. By evaluating the co-expression patterns of these markers in a comprehensive immunohistochemical panel, this study seeks to enhance the diagnosis and classification of SPNs, especially in challenging cases.

## Materials and methods

### Ethical approval

Ethical approval was obtained from the International Review Board of the National Cancer Institute, Cairo University, (Approval number PA2502 - 501–091–196, IRB number IRB00004025).

### Study design and population

This retrospective study includes 60 patients diagnosed with SPNs between January 2015 and November 2024. The cohort was drawn from institutional Oncologic Pathology archives, including both male and female patients across a broad age range. Four pediatric cases (ages 8–14 years) were included, providing a unique opportunity to explore the applicability of CD99 and LEF1 in diagnosing SPNs in children.

### Immunohistochemical analysis

Immunohistochemical analysis was conducted on formalin-fixed, paraffin-embedded (FFPE) sections cut at 4–5 µm. Tissue sections underwent standard deparaffinization in xylene and rehydration through a descending ethanol gradient. Antigen retrieval was achieved via heat-induced epitope retrieval using either citrate buffer (pH 6.0) or Tris–EDTA buffer (pH 9.0), selected according to the antigen target. Endogenous peroxidase activity was blocked with 3% hydrogen peroxide, and non-specific antibody binding was minimized using a protein blocking solution prior to incubation with primary antibodies. The primary antibodies utilized were CD99 (clone 12E7, Roche), LEF1 (clone 5E6, Ventana), Cyclin D1 (clone EP12, Roche), β-catenin (clone E- 5, DAKO), progesterone receptor (PR; clone 1E2, Ventana) and Ki- 67 (clone 30–9, Roche). All antibodies were applied at optimized dilutions and incubated either at room temperature or overnight at 4 °C. Detection was carried out using the avidin–biotin peroxidase method with diaminobenzidine (DAB) serving as the chromogen, followed by counterstaining with hematoxylin. Slides were then dehydrated, cleared, and mounted for microscopic evaluation. Internal quality controls included positive SPN cases for each antibody to confirm staining consistency and specificity, while negative controls were processed in parallel by omitting the primary antibody. Marker positivity was interpreted based on thresholds adapted from relevant literature and validated internally: CD99 was considered positive when ≥ 10% of tumor cells demonstrated cytoplasmic staining with paranuclear expression; β-catenin was deemed positive when ≥ 5% of tumor cells showed nuclear staining, with or without accompanying cytoplasmic signal; Cyclin D1 positivity was defined by ≥ 10% of tumor cells exhibiting moderate to strong nuclear staining; and PR expression was considered positive when ≥ 1% of tumor nuclei stained, in accordance with breast pathology standards due to the hormone receptor context. The Ki- 67 labeling index was determined by manually counting positively stained tumor cell nuclei in regions of highest proliferative activity, commonly referred to as"hot spots."A minimum of 500 neoplastic cells were evaluated per case at × 400 magnification. Only distinct nuclear staining was considered positive; cytoplasmic or ambiguous signals were excluded. Areas showing necrosis, crush artifact, or poor preservation were carefully avoided Additional immunohistochemical markers employed in pediatric cases for differential diagnosis included Chromogranin, CD56, BCOR, E-cadherin, DOG- 1, WT1, Desmin, Myogenin, LCA, TDT, FLI- 1, and NKX2.2. Findings for these ancillary markers were extracted from the original diagnostic pathology reports and confirmed through review of archived slides when available. Representative images of positive controls are provided to illustrate staining specificity and reproducibility across all targets.

### Statistical analysis

The data were analyzed using SPSS software (version 27). Descriptive statistics were calculated for tumor characteristics, including size, location, and patient age. Associations between immunohistochemical markers were evaluated non parametric tests. The significance level was set at *p* < 0.05.

## Results

### Demographics and tumor characteristics

The cohort comprised 60 patients with histologically confirmed solid pseudopapillary neoplasms (SPNs) of the pancreas. Of these, 52 (86.7%) were female and 8 (13.3%) were male, yielding a male-to-female ratio of nearly 1:7. The age range was 8 to 58 years, with a median of 32.5 years and a mean of 32.5 ± 12.6 years. Pediatric patients (age ≤ 18) accounted for 4 cases (6.7%), aged 8–14 years.

**Fig. 1:**
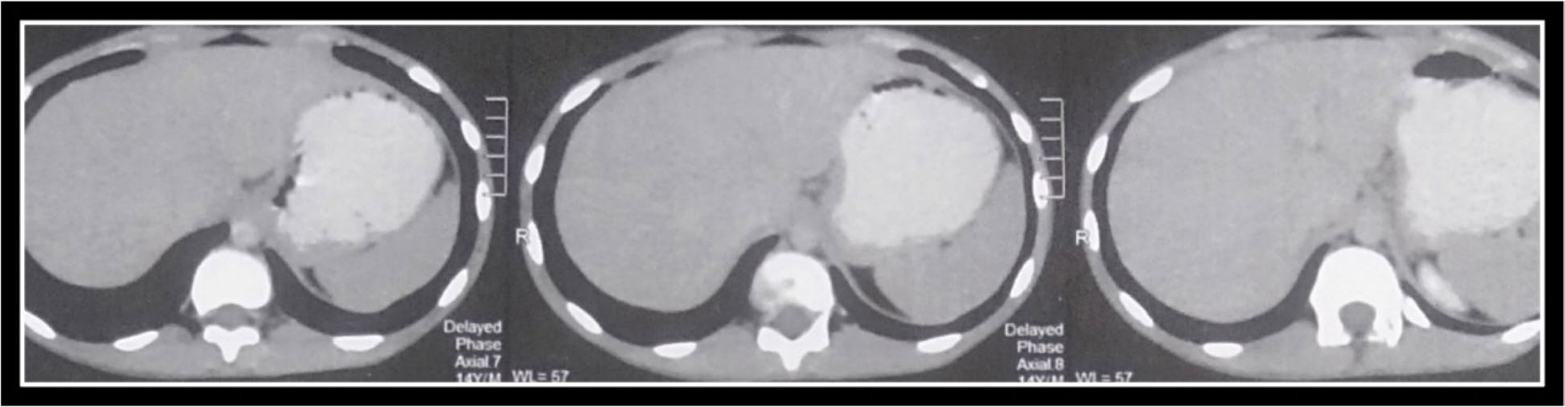
The imaging shows a well-defined, rounded, heterogeneous lesion in the pancreatic head. The mass measures approximately 8 cm in diameter, with heterogeneous post-contrast enhancement. Notable features include splaying of the portal vein posteriorly, without evidence of vascular invasion. These radiological findings are consistent with a solid pseudopapillary neoplasm

**Fig. 2:**
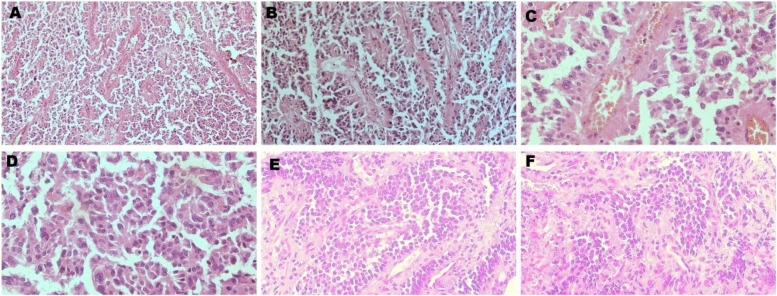
H&E stained sections of the pediatric case report **A** original magnification × 100, & **B**, **E**, **F** original magnification × 200, **C**&**D** original magnification × 400). The tumor shows a combination of solid and pseudopapillary growth patterns. Tumor cells are arranged around fibrovascular cores, demonstrating a uniform appearance with round-to-oval nuclei and eosinophilic cytoplasm. Evidence of hemorrhagic and cystic degeneration is present, which is characteristic of SPNs

Tumor sizes ranged from 2 to 12 cm, with a mean of 6.7 ± 2.5 cm and a median of 6.4 cm. The distribution of tumor sites included the pancreatic tail (*n* = 27, 45%), body (*n* = 21, 35%), and head (*n* = 12, 20%). Lymphovascular invasion (LVI) was present in 6 cases (10%). Figure 1 demonstrates radiological features of a male child aged 12 years. No cases demonstrated lymph node metastasis or perineural invasion. Figure 2 demonstrates histomorphololigical criteria of studied cases. Surgical procedures performed included distal pancreatectomy ± splenectomy (*n* = 25, 41.7%), pancreaticoduodenectomy (Whipple operation) (*n* = 22, 36.7%), and enucleation (*n* = 13, 21.6%).

### Immunohistochemical marker expression

**Fig. 3:**
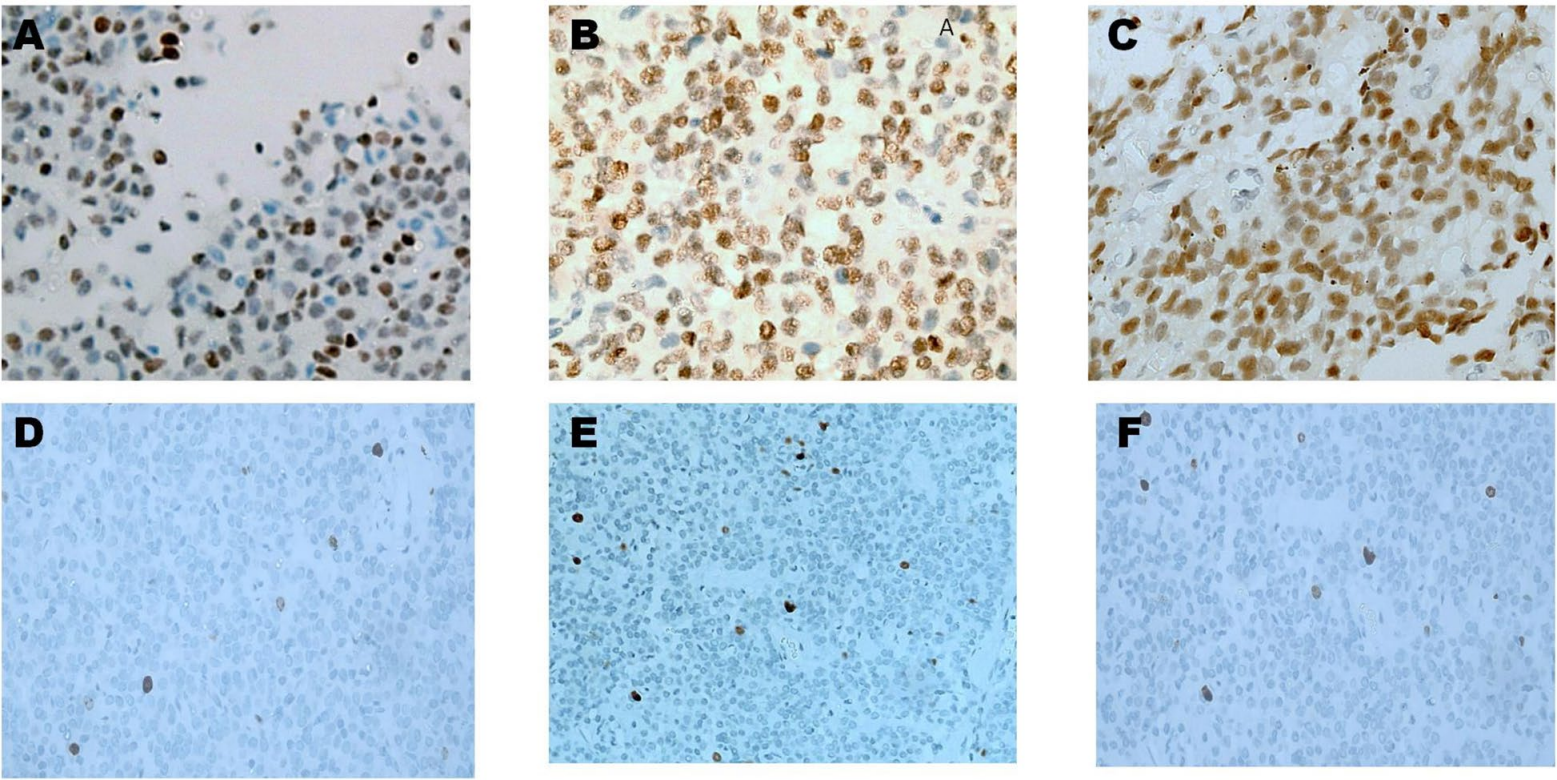
**A**,**B**,**C** Immunohistochemical staining to PR shows nuclear positivity of tumor cells,. Cytoplasmic or membranous staining is absent. (Original magnification × 200 (**A**&**C**), × 400(**B**)). **D**,**E** &**F**: Immunohistochemical staining to Ki- 67 shows nuclear positivity of scattered tumor cells,. (Original magnification × 200)

**Fig. 4:**
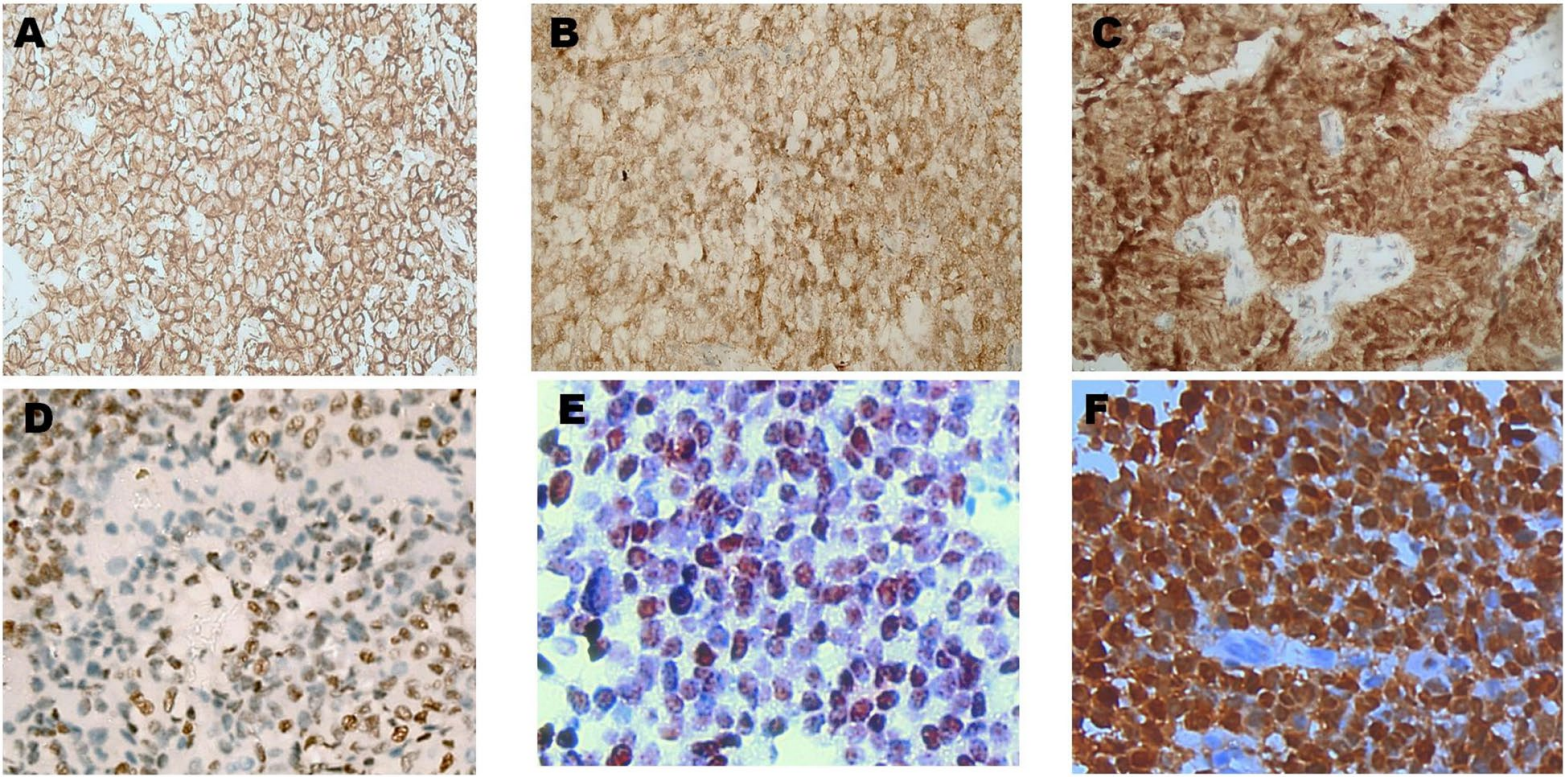
A. **B**, **C** Immunohistochemical staining with monocloncal antibody to β-catenin shows diffuse membranous staining of tumor cells in A (non specific negative reaction), combined nuclear and cytoplasmic expression in **B** & **C** (Original magnification × 100 (**A**), × 200(**B**&**C**). **D**, **E**, **F** Immunohistochemical staining to cyclin D1 shows nuclear staining of tumor cells, indicative of dysregulated cell cycle progression linked to β-catenin signaling. supporting the diagnosis of SPN. (Original magnification × 200 (**A**), × 400(**B**))

**Fig. 5:**
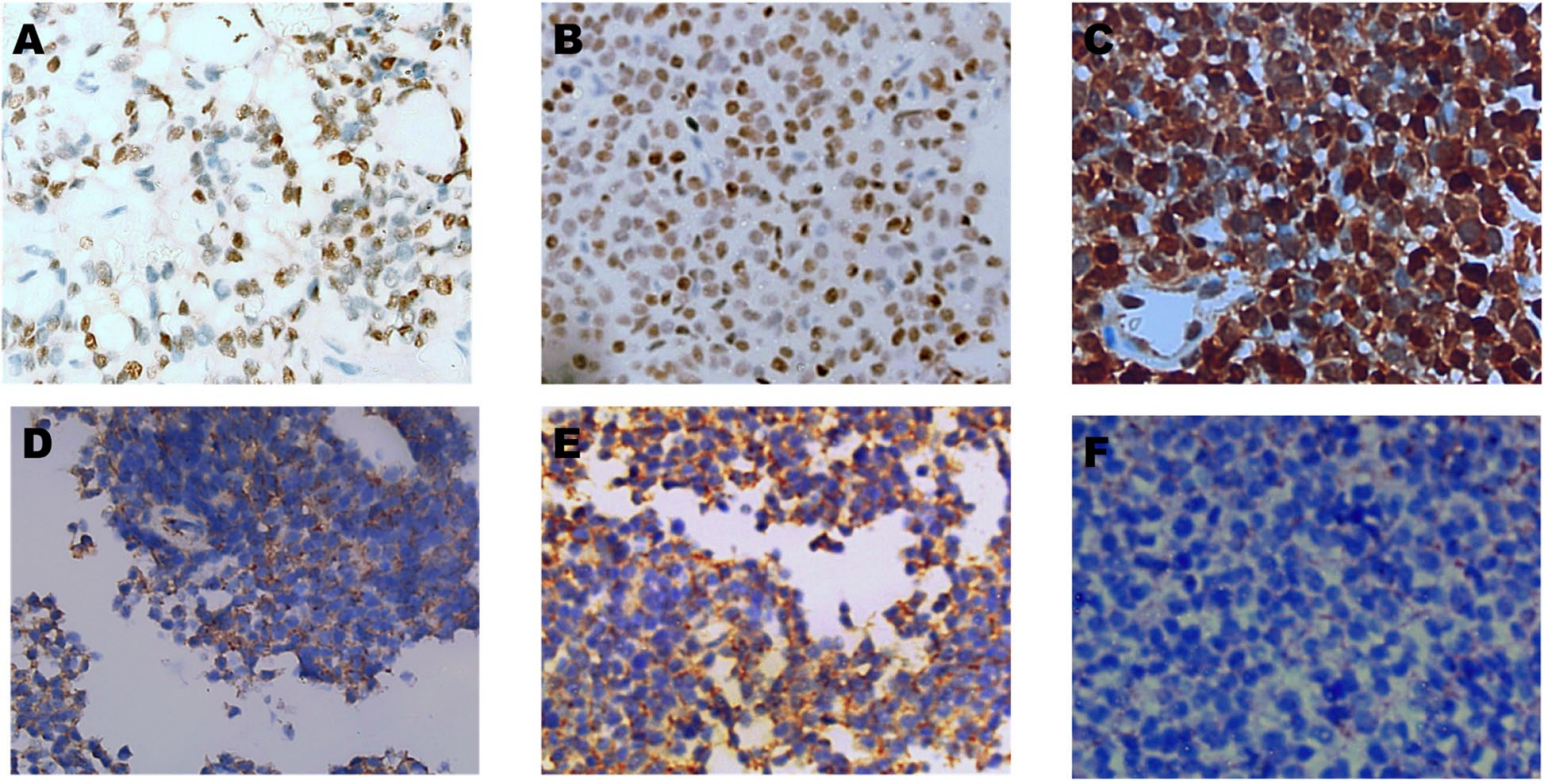
**A**,**B**,**C** Immunohistochemical staining to LEF1 shows nuclear staining of tumor cells, strongly supporting the diagnosis of SPN. Cytoplasmic staining is absent, aligning with the specific localization of LEF1 in SPNs. (Original magnification × 200 (**A**&**B**), × 400(**C**)). **D**, **E**, **F** Immunohistochemical staining for CD99 reveals positivity with para-nuclear accentuation in tumor cells, helping to differentiate SPNs from other pancreatic neoplasms. The staining intensity is consistent throughout the majority of the tumor, facilitating diagnostic distinction. (Original magnification × 200 (A&B), × 400 (**C**))

**Fig. 6:**
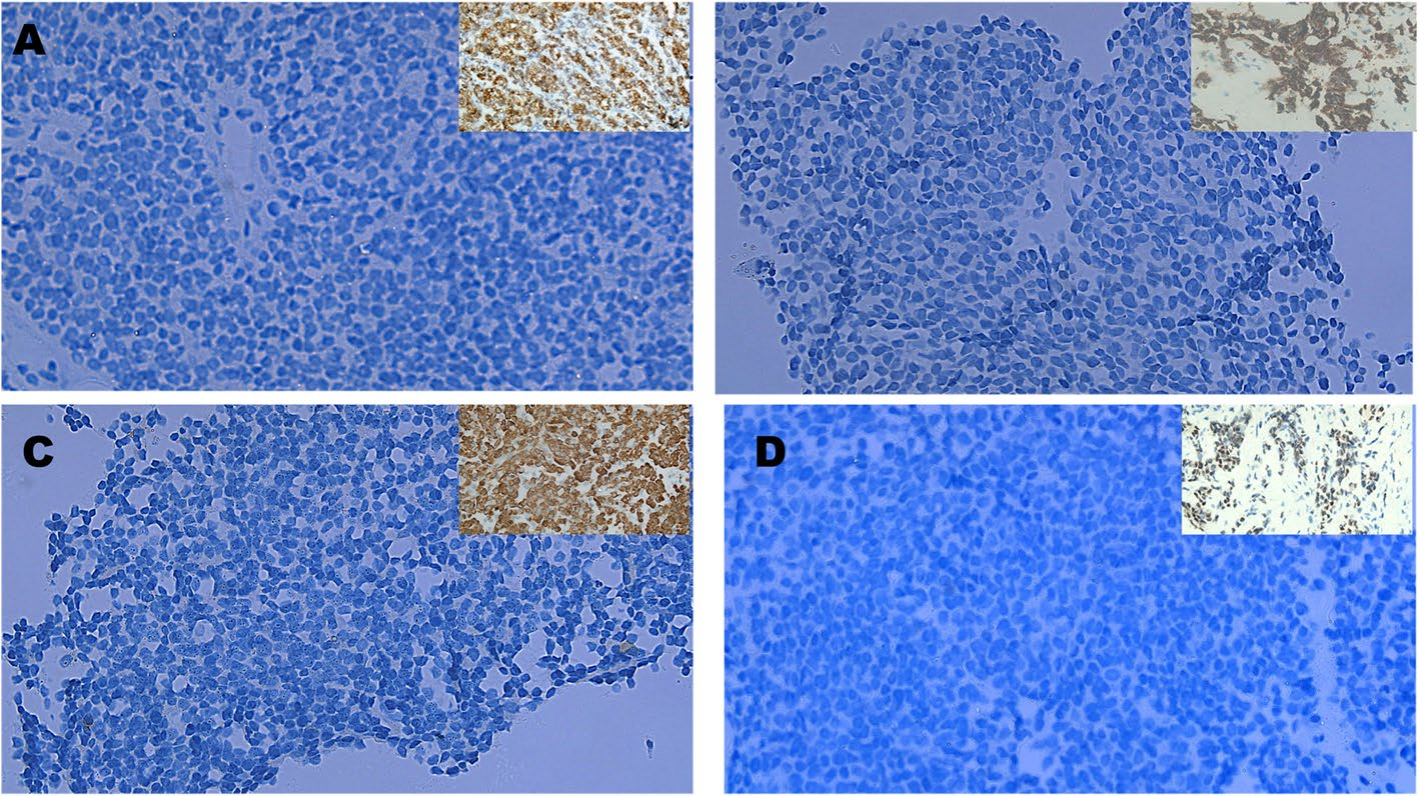
Immunohistochemical staining of pediatric solid pseudopapillary tumor (SPT) samples, all showing negative expression for the tested markers. **A** NKX2.2, **B** Chromogranin A, (**C**) Desmin, and (**D**) Myogenin immunolabeling revealed no detectable expression within tumor cells across all cases. Insets in each panel show external positive control tissues demonstrating appropriate marker reactivity, confirming assay validity. Original magnification: × 200

The expression of key immunohistochemical markers was quantified across 60 solid pseudopapillary neoplasms (SPNs), with the following results. The Ki- 67 Fig. 3 index had a mean of 4.1% (± 2.45), ranging from 2 to 9%. β-Catenin Fig. 4 was positive in 57 tumors (95%), with nuclear localization observed in ≥ 30% of tumor cells in all positive cases, yielding an average positivity of 84.2% (SD: 27.6). Progesterone Receptor (PR) Fig. 3 was detected in 53 cases (88.3%), with > 50% nuclear staining in most cases, resulting in a mean positivity of 62% (SD: 24.1). CD99 Fig. 5 was positive in 58 tumors (96.7%), with 55 cases showing diffuse cytoplasmic expression, accentuated by paranuclear dot-like staining. LEF1 Fig. 5 was positive in 55 tumors (91.7%), showing strong nuclear staining with a mean positivity of 75.7% (SD: 28.6). Cyclin D1 Fig. 4 exhibited nuclear positivity in 54 tumors (90%), with more than 50% of cells stained, averaging 33.6% positivity (SD: 15.8). Figure 6 demonstrates negativity for NKX2.2, chromogranin, desmin & myogenin -with external positive controls- that were performed in pediatric cohort.

### Marker coexpression

The coexpression patterns of these markers were as follows: 44 tumors (73.3%) demonstrated coexpression of β-catenin, PR, CD99, LEF1, and Cyclin D1. Six tumors (10%) expressed only β-catenin, CD99, and LEF1, while five tumors (8.3%) expressed β-catenin and LEF1 without PR or Cyclin D1. These findings suggest a predominant coexpression of multiple key markers, reflecting a robust molecular profile in most SPNs.

### Correlation with clinical and pathological parameters

**Fig. 7:**
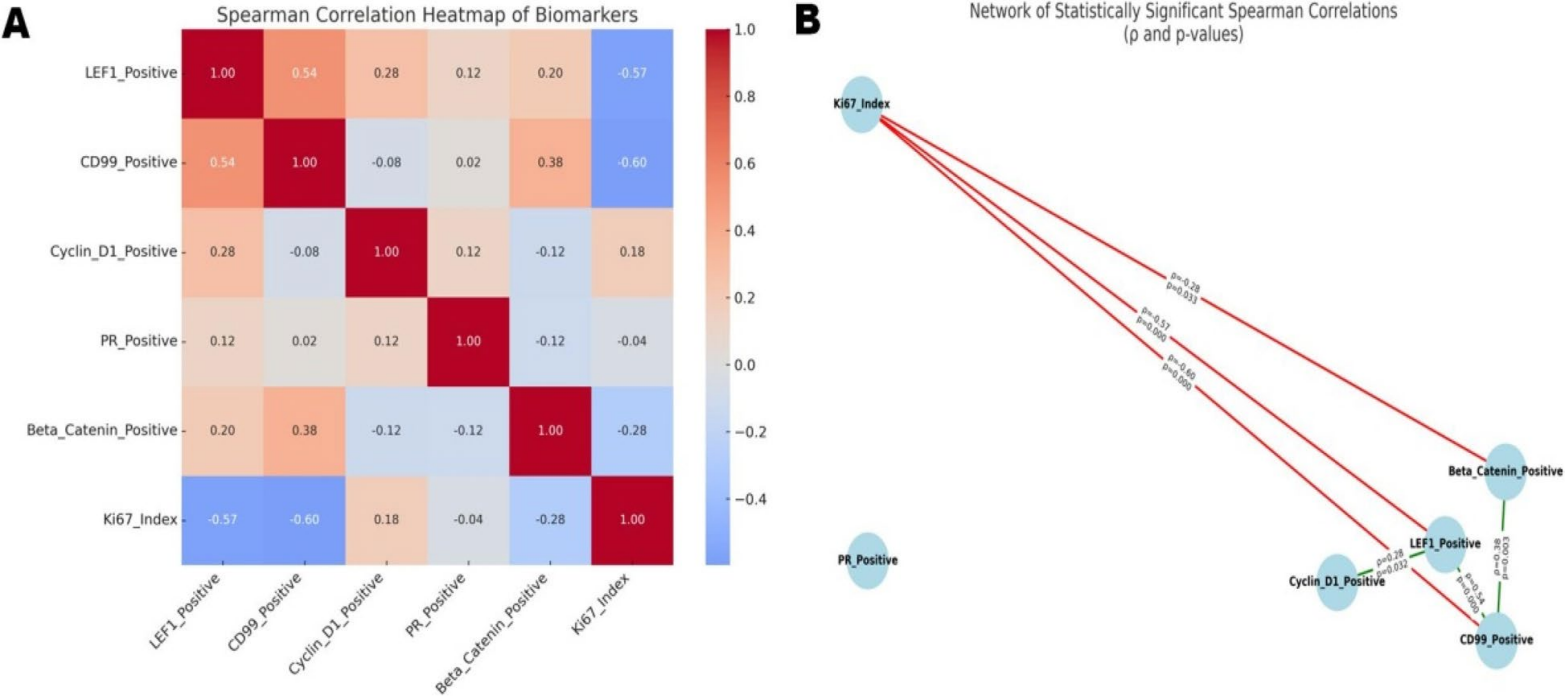
(**A**): Heatmap of Spearman’s rank correlation coefficients between six biomarkers: LEF1, CD99, Cyclin D1, PR, Beta-Catenin, and Ki67 Index. Positive correlations are shown in shades of blue, and negative correlations in red. Stronger relationships are indicated by more intense colors. **B** The network diagram illustrates statistically significant correlations (*p* < 0.05) between various biomarkers. Each node represents a specific biomarker, with edges connecting nodes that demonstrate significant associations. Green edges indicate positive correlations, while red edges signify negative correlations. The labels on the edges display both the correlation coefficient (ρ) and the corresponding p-value. This network visually emphasizes key relationships, particularly the strong inverse correlations between LEF1 and CD99 with the Ki- 67 index, suggesting that these markers may be indicative of lower cellular proliferation

Spearman correlation analysis revealed a moderate positive correlation between Cyclin D1 expression and T stage (*r* = 0.476, *p* < 0.01). Weak but significant correlations were observed between PR positivity and T stage (*r* = 0.254, *p* < 0.05), as well as between the Ki- 67 index and T stage (*r* = 0.297, *p* < 0.05). No significant correlation was found between tumor size and any biomarker. Regarding risk factors, PR expression did not correlate with patient sex (*p* = 0.15) or age group (*p* = 0.12), and tumor size was similar between PR-positive and PR-negative tumors (*p* = 0.27). Lymphovascular invasion (LVI)-positive cases had higher Ki- 67 indices (mean 7.0%) compared to LVI-negative cases (mean 3.76%, *p* = 0.045). Additionally, CD99 positivity was significantly higher in LVI-negative tumors (mean rank comparison, *p* = 0.023). The following Tables 1,  2, and 3 demonstrates detailed statistics regarding distribution of tumors specified by tumor location (i.e. pancreatic head, body or tail), T stage and presence or absence of lymphovascualar invasion. Table 4 demonstrates Non parametric tests for lymphovascular space invasion and immunohistochemical marker expression. Figure 7 demonstrates a heat map and statistical network for biomarker correlations.

**Table 1:** Descriptive statistics of tumor parameters by tumor location

**Variable**	**Tumor Location**	**Mean**	**Std. Deviation**	**Median**	**Range**	**Min–Max**	**IQR**	**95% CI (Lower–Upper)**
**Tumor Size (cm)**	Head	6.1	1.49	5.5	3.7	4.3–8.0	3.2	5.15–7.05
	Body	6.3	2.19	6.7	7.2	2.9–10.1	3.9	5.30–7.29
	Tail	7.44	3.01	6.65	10	2.0–12.0	5.9	6.23–8.66
**Ki67 Index (%)**	Head	3.67	1.56	3	4	2.0–6.0	3.3	2.68–4.66
	Body	3.33	1.85	3	6	2.0–8.0	2	2.49–4.18
	Tail	4.96	2.88	3	7	2.0–9.0	6	3.80–6.12
**LEF1 Positive (%)**	Head	87.58	6.83	85.5	20	80.0–100.0	10.8	83.25–91.92
	Body	79.43	27.74	88	99	0.0–99.0	15	66.80–92.06
	Tail	65.89	31.75	73.5	100	0.0–100.0	46.5	53.06–78.71
**CD99 Positive (%)**	Head	61.92	7.91	62	24	51.0–75.0	11.8	56.89–66.95
	Body	59.57	18.47	60	81	0.0–81.0	22.5	51.16–67.98
	Tail	53.19	19.97	52	96	0.0–96.0	30.5	45.13–61.26
**Cyclin D1 Positive (%)**	Head	39.58	9	39.5	29	22.0–51.0	14	33.87–45.30
	Body	29.71	17.76	35	50	0.0–50.0	25	21.63–37.80
	Tail	34.46	14.1	39.5	50	0.0–50.0	10	28.77–40.16
**PR Positive (%)**	Head	71.33	7.63	72.5	22	60.0–82.0	15.3	66.48–76.18
	Body	66.19	16.11	70	77	0.0–77.0	11	58.86–73.53
	Tail	54.12	30.73	68	85	0.0–85.0	27.5	41.70–66.53
**β-Catenin Positive (%)**	Head	87.67	27.71	94.5	100	0.0–100.0	4.5	70.06–105.27
	Body	90.33	20.91	94	100	0.0–100.0	4.5	80.82–99.85
	Tail	77.19	34.7	94	100	0.0–100.0	23	63.18–91.21

**Table 2:** Descriptive statistics of tumor parameters by tumor stage

Variable	T Stage	Mean	Std. Dev	Median	Min	Max
Tumor_Size_cm	2	3.48	0.39	3.55	2.9	4
	3	7.24	2.34	6.8	4.2	12
	4	7.83	1.32	7.95	6.5	10.1
Ki67_Index	2	2.38	0.52	2	2	3
	3	4.4	2.53	3	2	9
	4	4.33	2.25	3.5	2	8
LEF1_Positive	2	66.63	41.51	85.5	0	95
	3	78.93	21.5	86	0	100
	4	72.83	36.03	85.5	0	93
CD99_Positive	2	51.75	33.2	64	0	81
	3	57.53	13.31	57	30	79
	4	58.17	15.59	55.5	35	78

N.B: Statistical measures were not computed for T Stage 1 due to the presence of only a single data point (*N* = 1), which does not allow for meaningful statistical analysis

**Table 3:** Descriptive Statistics by Lymphovascular Invasion (LVI) Status

Marker	LVI*	N (Valid)	Mean	SD	Median	Min	Max	Range	95% CI (Lower–Upper)
**Tumor Size (cm)**	0	54	6.42	2.44	6.1	2	12	10	5.76–7.09
	1	6	9.32	1.81	10.2	6.5	10.8	4.3	7.42–11.21
**Ki67 Index** (%)	0	54	3.76	2.21	3	2	9	7	3.16–4.36
	1	6	7	2.19	8	3	9	6	4.70–9.30
**LEF1 Positive** (%)	0	54	77.65	26.47	87	0	100	100	70.42–84.87
	1	6	54.83	34.25	63	0	93	93	18.89–90.78
**CD99 Positive** (%)	0	54	58.63	17.66	59.5	0	96	96	53.81–63.45
	1	6	47	15.59	45	30	73	43	30.63–63.37
**Cyclin D1 Positive** (%)	0	54	32.76	16.11	37	0	51	51	28.36–37.15
	1	6	37.67	6.02	36.5	32	48	16	31.35–43.99
**PR Positive** (%)	0	54	63.35	21.25	69.5	0	82	82	57.55–69.15
	1	6	49.67	38.86	70.5	0	85	85	8.89–90.45
**β-Catenin Positive** (%)	0	54	84.33	28.83	94	0	100	100	76.46–92.20
	1	6	83.33	33.56	97	15	100	85	48.11–118.55

0 indicates absence of LVI, 1 indicates presence of LVI

**Table 4:** Non parametric tests for lymphovascular space invasion and immunohistochemical marker expression

**Biomarker**	**Spearman’s Correlation** **Coefficient** **(ρ)**	**Significance (*p*-value)**	**Interpretation**	Partial correlation **Coefficient (ρ)**	***p***-value
Lymphovascular invasion	**0.342**	**0.008**	**Moderate positive association; suggests that larger tumors may have a greater propensity for lymphatic or vascular invasion.**	0.348	0.007
Ki- 67 Index	**0.735**	***p* < 0.001**	**Strong positive correlation with tumor size; suggests that larger tumors exhibit significantly higher proliferative activity.**	0.833	< 0.001
Cyclin D1 Positive (%)	**0.415**	***p* = 0.001**	**Moderate-to-strong positive correlation; larger tumors show increased Cyclin D1 expression, indicating active cell cycle progression**	0.548	< 0.001
CD99 Positive (%)	**–0.499**	***p* < 0.001**	**Moderate negative correlation; CD99 is more highly expressed in smaller or early-stage tumors, possibly indicating a role in tumor suppression.**	–0.359	< 0.01
LEF1 Positive (%)	**–0.326**	***p* = 0.011**	**Weak negative correlation; LEF1 may be associated with less aggressive tumor phenotypes or early developmental stages.**	–0.245	0.064
β-Catenin Positive (%)	–0.151	*p* = 0.250	Not statistically significant; minimal or inconsistent relationship with tumor size in this cohort.	–0.227	0.087
PR Positive (%)	–0.016	*p* = 0.902	No significant association; PR expression appears independent of tumor size.	–0.231	0.081

Comment: Spearman’s correlation analysis demonstrated a strong positive relationship between the Ki- 67 proliferation index and tumor size (*r* = 0.735, *p* < 0.01), implying that increased tumor dimensions are associated with heightened proliferative activity. A moderate correlation was also observed between Ki- 67 and lymphovascular invasion (*r* = 0.368, *p* < 0.01), suggesting that proliferative tumors may have a greater tendency for vascular or lymphatic infiltration. Similarly, tumor size correlated moderately with lymphovascular invasion (*r* = 0.342, *p* < 0.01), further indicating a potential association between tumor burden and invasive potential. Consistent with these findings, Cyclin D1 expression positively correlated with tumor size, reinforcing the link between tumor growth and enhanced cell cycle progression. Conversely, CD99 and LEF1 expression demonstrated inverse associations with tumor size, suggesting their relevance may be confined to smaller or less aggressive neoplasms. When controlling for age group (children vs. adults) through partial correlation analysis, the associations between tumor size and both Ki- 67 (*r* = 0.833, *p* < 0.01) and Cyclin D1 (*r* = 0.548, *p* < 0.01) remained robust, supporting the role of these markers in proliferative expansion. Negative correlations persisted between tumor size and CD99 (*r* = –0.359, *p* < 0.01) and LEF1 (*r* = –0.245, *p* = 0.064), reinforcing their possible involvement in earlier or less advanced disease stages. Additionally, Ki- 67 was negatively associated with both CD99 and LEF1, which may reflect their potential inhibitory influence on proliferative activity. Cyclin D1 also showed positive correlations with T stage and clinical symptoms, indicating its relationship with more advanced or symptomatic tumors. In contrast, β-Catenin and progesterone receptor (PR) expression exhibited weak or non-significant associations with tumor size and other clinical parameters. The association between lymphovascular invasion and both Ki- 67 and tumor size appeared modest and was not consistently observed across all markers evaluated

### Pediatric vs. adult comparison

Among the pediatric cases (*n* = 4), there were three females and one male. Tumor sizes ranged from 4.5 cm to 8.0 cm, with the largest lesion observed in the male patient. Moreover, the pediatric male case exhibited negative nuclear expression to beta-catenin. Pediatric tumors demonstrated slightly higher mean expression for PR (70.5% ± 6.2 vs. 61.4% ± 24.1), LEF1 (86.5% ± 7.5 vs. 74.6% ± 28.6), and Cyclin D1 (41% ± 7 vs. 32.7% ± 15.8) than adult tumors. Ki- 67 indices were comparable (3.5% vs. 4.1%). Notably, CD99 positivity was higher in pediatric cases (mean 73.8% ± 6.4) compared to adults (56.3% ± 17.7). Table 5 demonstrates a detailed comparison between adult and pediatric cohorts as regards tumor size and marker expression profile. None of the pediatric tumors exhibited lymph node metastasis; however, one case demonstrated lymphovascular invasion (LVI). In contrast, five adult cases were found to have lymphovascular invasion .

**Table 5:** Comparison between adult and pediatric cohorts as regards tumor size and marker expression profile

Variable	Group	N	Mean	Std. Deviation	Median	Min	Max	Range	25 th Percentile	75 th Percentile
**Tumor Size (cm)**	Children	4	6.775	1.6132	7.25	4.6	8	3.4	5.075	8
	Adults	56	6.707	2.5906	6.25	2	12	10	4.525	9.425
**Ki67 Index**	Children	4	3.5	1.7321	3	2	6	4	2.25	5.25
	Adults	56	4.125	2.45	3	2	9	7	2	6
**LEF1 Positive (%)**	Children	4	86.5	7.5056	86.5	80	93	13	80	93
	Adults	56	74.571	28.6356	85.5	0	100	100	65.25	92.75
**CD99 Positive (%)**	Children	4	73.75	6.3966	75.5	65	79	14	67	78.75
	Adults	56	56.304	17.6933	56	0	96	96	50	67.75
**Cyclin D1 Positive (%)**	Children	4	41	6.976	39	35	51	16	35.75	48.25
	Adults	56	32.7	15.756	36.5	0	50	50	31	42
**PR Positive (%)**	Children	4	70.5	6.245	71.5	62	77	15	64.25	75.75
	Adults	56	61.375	24.1439	69.5	0	85	85	62.25	74
**Beta-Catenin Positive (%)**	Children	4	72.75	48.5618	95.5	0	100	100	23.5	99.25
	Adults	56	85.054	27.6507	94	0	100	100	92	97.75

## Discussion

Solid pseudopapillary neoplasm (SPN) of the pancreas represents a rare and enigmatic pancreatic tumor. Despite its relatively low incidence, accounting for approximately 1–2% of all pancreatic neoplasms, SPN has garnered significant attention due to its distinctive clinical, histopathological, and molecular characteristics, as well as its generally favorable prognosis following surgical resection, [15]. However, its differential diagnosis and the molecular pathways driving tumorigenesis remain complex and require a nuanced understanding. In this study, we aim to extend current knowledge by providing an in-depth analysis of the immunohistochemical markers, the pivotal role of the Wnt/β-catenin signaling pathway, and their implications for clinical prognosis and therapeutic approaches.

The predominance of SPNs in young women, as confirmed in our study, mirrors the demographic profile consistently reported in global studies [6–8]. The observed increased female-to-male ratio and the mean age of presentation in the third decade of life suggest hormonal influence in the pathogenesis of SPNs, supported by the expression of progesterone receptors (PR) in most cases [15–19]. However, our inclusion of male and pediatric cases highlights the necessity of considering SPNs in atypical demographic groups. This is particularly significant given prior studies, such as those by Wu et al. that report biologically aggressive behavior in male patients [18]. Our findings align with this observation, as the pediatric male case in our study presented with a relatively large tumor (8 cm in diameter), reflecting the broader spectrum of SPN presentations [20, 21].

Accurate pathological evaluation is critical for distinguishing solid pseudopapillary tumors (SPTs) from other pancreatic neoplasms with overlapping features. Histologically, SPTs exhibit a characteristic combination of solid and pseudopapillary patterns, often accompanied by cystic and hemorrhagic degeneration [6, 16]. In our study, all cases demonstrated this hallmark architecture, including uniform polygonal cells with ovoid nuclei, nuclear grooves, and occasional eosinophilic hyaline globules. Given these morphological features, the differential diagnosis primarily included pancreatic neuroendocrine tumors (PanNETs) and acinar cell carcinomas (ACCs). However, immunohistochemistry played a pivotal role in confirming the diagnosis. All SPT cases were negative for neuroendocrine markers such as chromogranin A and synaptophysin but showed strong nuclear positivity for β-catenin in most cases, indicative of aberrant Wnt/β-catenin signaling. This immunophenotypic profile aligns with previous studies identifying nuclear β-catenin as a defining diagnostic feature of SPTs and absent in most PanNETs and ACCs [22, 23]. These findings underscore the necessity of integrating histological and immunohistochemical criteria for the accurate diagnosis of SPTs.

The molecular pathogenesis of SPN is predominantly driven by mutations in the CTNNB1 gene, which encodes β-catenin, a key regulator in the Wnt/β-catenin signaling pathway. This pathway plays a fundamental role in controlling cell proliferation, differentiation, and survival, making its dysregulation a critical event in cancer development. In SPNs, mutations in CTNNB1 result in the stabilization of β-catenin, which accumulates in the cytoplasm and translocates to the nucleus, where it activates the transcription of target genes such as Cyclin D1, c-Myc, and other key cell cycle regulators. Our findings support this model, as we observed nuclear β-catenin positivity in 95% of SPN cases, underscoring the ubiquitous nature of this molecular alteration in SPNs. The aberrant nuclear accumulation of β-catenin is not only a diagnostic feature but also a pivotal step in SPN tumorigenesis. The stabilization of β-catenin results from mutations that impair its degradation, leading to unchecked activation of the canonical Wnt signaling pathway. This, in turn, drives the transcriptional activation of Cyclin D1, a cyclin-dependent kinase regulator that promotes the G1-S phase transition in the cell cycle, thereby facilitating tumor growth [7, 10, 24].

While the CTNNB1 mutation remains the most significant molecular alteration in SPNs, the complete genetic landscape of these tumors is far from fully elucidated. Other genetic events, such as mutations in genes involved in chromatin remodeling, cell cycle regulation, and tumor suppression, may also contribute to SPN pathogenesis, although their exact roles remain under investigation. Notably, mutations in the PIK3 CA gene, which encodes a subunit of phosphoinositide 3-kinase, have been identified in some SPNs, suggesting that alterations in the PI3 K-AKT signaling pathway may play a role in tumor development [25–28]

Our results presented the diagnostic utility of CD99 and LEF1, with expression rates of 96.7% and 91.7%, respectively. CD99, a cell surface glycoprotein, exhibited cytoplasmic positivity with paranuclear accentuation, aiding in distinguishing SPNs from pancreatic neuroendocrine tumors and other mimics [17, 27]. LEF1, a transcription factor in the Wnt/β-catenin pathway, showed strong nuclear positivity in most cases, further supporting its utility as a diagnostic marker [4, 28]. These findings are consistent with studies by Estrella et al. and Fujii et al., which similarly demonstrated the reliability of these markers in SPN diagnosis [21, 28].

Aberrant nuclear localization of β-catenin, is a hallmark molecular alteration in SPNs, driven by mutations in the CTNNB1 gene [29, 30]. This pathway’s role in SPN tumorigenesis has been well-documented in prior studies and is corroborated by our findings [21, 27–29]. The positive correlation between β-catenin and Cyclin D1 expression in our cohort suggests that dysregulation of this pathway significantly contributes to SPN proliferation and progression. Similar associations have been reported by Yang et al., who demonstrated that Cyclin D1 expression correlates with increased tumor size and proliferative capacity [28].

SPNs are generally considered indolent tumors with an excellent prognosis following complete surgical resection [6, 8]. However, male gender and large tumor size have been associated with aggressive behavior [18, 30, 31]. The pediatric male case in our study underscores this potential for biological aggressiveness in atypical presentations, as the tumor exhibited rapid growth, necessitating aggressive surgical intervention. These findings align with studies by Tang et al., who reported similar patterns in aggressive SPN variants [32].

Surgical resection remains the gold standard for SPN management, with procedures such as pancreaticoduodenectomy and distal pancreatectomy achieving high survival rates [6, 19]. Recent advancements in minimally invasive techniques, such as laparoscopic enucleation, have expanded the surgical options for small, localized SPNs [30]. Our study corroborates these findings. Imaging modalities, including contrast-enhanced CT and MRI, have further facilitated preoperative planning by reliably identifying SPNs based on their cystic and solid components [30, 31, 33].

## Conclusion

This study reinforces the distinctive immunophenotypic and molecular landscape of solid pseudopapillary neoplasms of the pancreas, highlighting the central role of the Wnt/β-catenin signaling axis in their pathogenesis. The high expression rates of CD99 and LEF1, alongside β-catenin and Cyclin D1, underscore the diagnostic reliability of this panel in confirming SPNs, particularly in histologically equivocal or demographically atypical cases such as males and pediatric patients. The observed coexpression patterns and correlations with tumor size, proliferation index, and lymphovascular invasion offer further insight into the biological behavior of these tumors.

## Data Availability

Data sets are available upon reasonable request from the corresponding author.
